# An Explanatory Model of the Relationships between Physical Activity, Social Support and Screen Time among Adolescents

**DOI:** 10.3390/ijerph19127463

**Published:** 2022-06-17

**Authors:** Daniel Sanz-Martín, Eduardo Melguizo-Ibáñez, Germán Ruiz-Tendero, José Luis Ubago-Jiménez

**Affiliations:** 1 Faculty of Humanities and Social Sciences, Isabel I University, 09003 Burgos, Spain; daniel.sanz6718@ui1.es; 2 Department of Didactics Musical, Plastic and Corporal Expression, Faculty of Education Science, University of Granada, 18071 Granada, Spain; jlubago@ugr.es; 3 Department of Languages, Arts and Physical Education Teaching, Faculty of Education, Complutense University of Madrid, 28040 Madrid, Spain; german.ruiz@edu.ucm.es

**Keywords:** physical activity, social support, screen time, adolescent, gender

## Abstract

Effective physical activity studies are necessary to understand how factors involved in physical activity mediate behaviour. Therefore, more reliable explanatory models can be generated in order to design effective actions to promote physical activity. The study had two aims: (1) to develop an explanatory model to identify and establish the relationships between physical activity, social support and screen time among adolescents; and (2) to contrast the explanatory structural model by means of a multi-group analysis according to sex. The study design was cross-sectional with descriptive and correlational analysis. The research was carried out with a representative sample of adolescents from the province of Soria (mean age 14.06 ± 1.27 years). The instruments used were: Four by One-Day Physical Activity Questionnaire, Parent Support Scale and Peer Support Scale. The results show that social support had a negative relationship with screen time (r = −0.178; *p* ≤ 0.001); males had a positive relationship between physical activity and screen time (r = 0.118; *p* ≤ 0.05); and peer support had more influence on social support than parental support. In conclusion, the proposed model was effective in triangulating the relationships between physical activity, social support and screen time in a novel way, while allowing us to discriminate these results according to participants’ sex.

## 1. Introduction

The practice of physical activity (PA) brings many physiological, psychological and social health benefits [1,2], such as a reduction in risk of cardiovascular disease [3] and cancer [4].

PA practice should be in accordance with international recommendations. In children and adolescents, this should be at least 60 min/day of moderate to vigorous physical activity, including aerobic exercise and bone-strengthening activities [5].

However, despite the benefits of PA, levels of practice in the global population are lower than recommended. For example, more than 70% of children and adolescents do not perform at the minimum recommended level [6,7]. Furthermore, PA levels have declined during the COVID-19 pandemic, and the situation has worsened [8].

In order to understand the current problem in depth, and, therefore, to be able to reverse the high level of physical inactivity, it is necessary to know the determinants that influence it and how they do so [9]. Among the most important correlates of adolescent PA are sex, support from parents and significant others, sedentary behaviour after school and sedentary behaviour on weekends [10].

Females engage in less PA than males [6,11], and PA decreases with increasing age [12,13].

### 1.1. Physical Activity and Social Support for Physical Activity

Social support (SS) is understood as “a network of family, friends, neighbours and community members that is available in times of need to give psychological, physical, and financial help” [14]. Parental and peer support have the greatest influence on adolescents’ PA [15]. Males receive higher levels of parental and peer support than females [16,17,18]. According to age, children’s perceived support from family members is higher than that from peers [17], but in adolescents, support from friends is higher [16,19].

According to a study by Mendonça et al. [15], there is no clear predominance regarding the relationship between adolescent PA and SS, since not all studies identified significant relationships, but it was confirmed that in the majority, there is a positive relationship between both variables, regardless of the sex of the participant [9,15,18,20,21,22]. Another study confirmed the positive and significant relationship between SS and compliance with PA recommendations in 74 countries [23]. 

### 1.2. Physical Activity and Screen Time

Sedentary Behaviour Research Network [24] defines sedentary behaviour as “any waking behaviour characterised by an energy expenditure ≤ 1.5 metabolic equivalents (METs), while in a sitting, reclining or lying posture”. This includes, for example, playing computer games, video games or watching television [25], which are known as screen time (ST) [26], or sedentary activities in front of a screen.

Although it is recommended for adolescents to spend no more than two hours per day on recreational screen time and to avoid prolonged continuous screen time [27], the levels found are higher than those recommended [28,29,30,31]. It would be advisable to reduce screen time and increase physical activity to protect young people’s mental health [3,20].

The relationship between PA and ST is negative [29,32,33]. Da Costa et al. [34] found that video viewing was related to lower levels of light and moderate to vigorous PA. According to gender, playing videogames in boys was associated with greater sedentary behavior and, in the case of girls, it was associated with the use of social networks. In a study with a Spanish adolescent population [35], no relationship was found between different screen behaviours and the level of PA, analysed independently, but the combination of several of them was negatively associated.

### 1.3. Social Support for Physical Activity and Screen Time

No scientific evidence has been found regarding the relationship between SS for PA and ST. Despite this, there are some studies that relate ST to other social variables.

Lucena et al. [36] found a positive relationship between “SS and peer group” and ST. The measurement of the social variable was with the KIDSCREEN-27 instrument, which asks about SS but in a generalized way, with no specific item on PA.

Zhang et al. [37] postulated that screen time was related to the type of general parenting. The relationship was indirect between ST and warmth, as well as with autonomy granting. In contrast, there was a positive relationship with coercive control.

Wong et al. [38] considered that adolescent ST was negatively and significantly related to family socioeconomic status. 

However, despite such evidence, no explanatory model has been found for the relationships between these variables in combination and directly measured. SS could be the most important determinant of the model [22], although more research is needed [9]. Models could make it easier to design health promotion interventions more efficiently for this population group [22,39,40].

**Figure 1:**
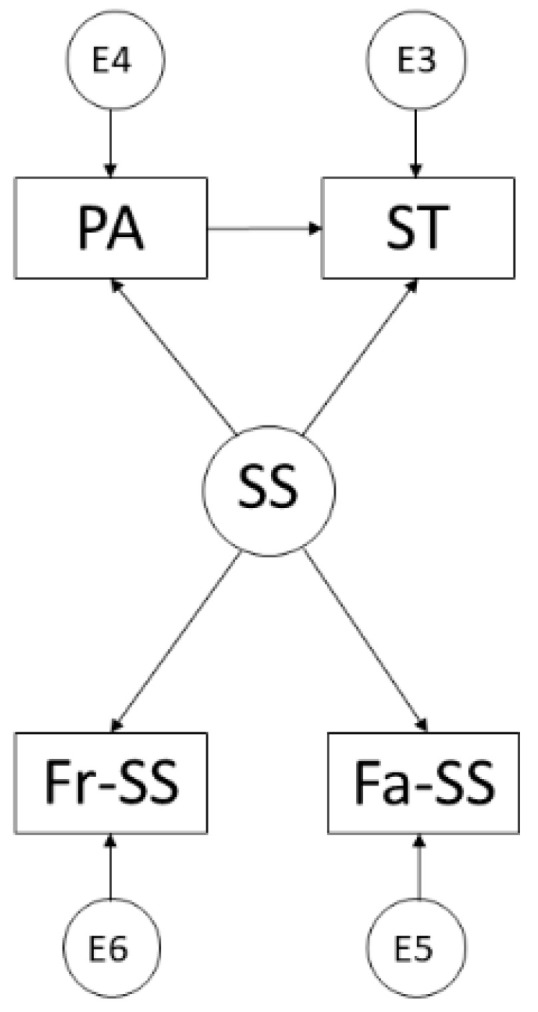
Proposed theoretical model. Note: Physical Activity (PA); Social Support (SS); Screen Time (ST); Family Support (Fa-SS); Friends’ Support (Fr-SS).

In view of the above, the aims of the present study were: (1) to develop an explanatory model to identify and establish the relationships between PA, SS and ST among adolescents and, (2) to contrast the explanatory structural model by means of a multi-group analysis according to sex. Accordingly, the theoretical model proposed was the one shown in Figure 1.

## 2. Materials and Methods

### 2.1. Design and Subjects

The study design was cross-sectional with descriptive and correlational analysis based on levels of PA, SS and ST.

The study population consisted of 3224 adolescents in compulsory secondary education in Soria (Spain), aged 12–17 years (14.06 ± 1.27). A non-probabilistic convenience sampling study was carried out, obtaining a sample of 694 students, which represented an error of precision of 3.3% for a confidence level of 95% and a standard deviation of 50. The sample consisted of 364 males (52.4%) and 330 females (47.6%). Moreover, 169 (24.4%) were first-year students, 179 (25.8%) were second-year students, 165 (23.8%) were third-year students and 181 (26.1%) were fourth-year students. 

### 2.2. Instruments and Variables

To measure PA levels, ST and SS, three instruments were used: Four by One-Day Physical Activity Questionnaire, Parent Support Scale and Peer Support Scale. The instruments were assessed together.

#### 2.2.1. Physical Activity Questionnaire

The Four by One-Day Physical Activity Questionnaire was designed and validated by Cale in a British adolescent population [41]. However, for the present study, we used the version by Cantera [42] adapted to Spanish adolescents and validated by Soler et al. [43]. 

This questionnaire is administered over four days and asks about the PA of the previous day. It asks about the PA performed on a school day when the young people have physical education classes, and on another day when they do not. Additionally, the questionnaire is administered on two Mondays to ask about PA performed on a Saturday and a Sunday. In addition, there are two questionnaire formats, one or the other being used depending on the day of the week (school day or weekend). 

This instrument provides socio-demographic information (sex and age) and information on PA (type of activity, duration and energy expenditure). It also allows recording the mode and ST, as several items request information on “watching TV” and use of “computer, video games and internet”. The reliability of the questionnaire is α = 0.832. This instrument has been used previously in other studies [44,45,46,47].

#### 2.2.2. Social Support Scales

Parent Support Scale and Peer Support Scale were designed by Prochaska et al. [48], but the Spanish version [46] was used. Each consists of five items on a Likert scale of 0–4 (0 = never; 4 = every day). Each asks about perceived support for PA during the last week and allows measuring family, peer and total environment support. 

The items of the scale for parents are: (1) parent encourages adolescent to engage in PA or sports, (2) parent engages in PA or sports with adolescent, (3) parent provides transportation to PA setting, (4) parent watches adolescent engage in PA or sports and, (5) parent tells adolescent she or he is doing well in PA or sports.

The items of the peer support scale are: (1) adolescent encourages friends to engage in PA or sports, (2) friends encourage adolescent to engage in PA or sports, (3) friends engage in PA or sports with adolescent, (4) peers tease adolescent for not being good in PA or sports and, (5) friends tell adolescent she or he is doing well in PA or sports.

The average score of each scale is calculated by adding all the points, except item 4 of the peer scale, which subtracts points, and dividing by the number of items in the scale. Regarding the reliability of the data, Cronbach’s alpha values range from 0.7–0.83. These scales have already been used in children [17] and adolescents [49].

### 2.3. Procedure

Firstly, a literature review was carried out to establish the state of the art in the scientific literature on the issue. The research project was prepared and presented to the Director and the Chief of the Education Office of the Provincial Department of Education in Soria, obtaining the required permission. Afterwards, permission was also obtained from the management team leaders to access the schools. Subsequently, an informed-consent form was given to the legal guardians of each student, which was returned signed so that the student could participate in the research.

After obtaining permission, the researchers proceeded to administer the assessment instruments to the adolescents in situ. The ethical principles established by the Declaration of Helsinki for research involving human subjects were followed. In addition, the present research was approved by the Ethics Committee of the University of Granada (1478/CEIH/2020).

On the day the researchers accessed the schools, they administered the paper questionnaires. There was at least one researcher for every six students, as established in the PA questionnaire protocol. The average response time was approximately 25 min/day. The reviewers were responsible for administering the instruments, explaining them, answering questions and checking that they had been correctly completed. 

### 2.4. Data Analysis

For descriptive analysis, the IBM SPSS Statistics 25.0 statistical programme (IBM Corp., Armonk, NY, USA) was used to perform means and standard deviation of the variable measures. The Kolmogórov–Smirnov test was used to determine the sample’s normality, obtaining a normal distribution. Finally, the Cronbach’s Alpha test was used to determine the reliability of the different instruments used.

In addition, the IBM SPSS Amos 26.0 program (IBM Corp, Armonk, NY, USA) was used to develop the structural equation models and, therefore, to study the relationships between the variables making up the theoretical model. A general model was applied for the whole sample and two models according to participants’ sex. The model was made up of a total of four observed or endogenous variables and one unobserved or exogenous variable. For endogenous variables, a causal explanation was obtained, taking into account the observed associations between the reliability of the measurement and the indicators. Therefore, the measurement error was included for the endogenous variables. Likewise, unidirectional arrows represented the influence lines between the latent variables, which were interpreted from the regression weights.

The proposed model fit was finally assessed. According to the proposed indications [50,51], the goodness-of-fit had to be evaluated on Chi-Square, where *p*-values and non-significant values indicated a correct fit model. In addition, it was indicated that the comparative fit index (CFI) should be above 0.95, the normal fit index (NFI) should reflect a score above 0.90, the incremental reliability index (IFI) should score above 0.90, and the root mean square error of approximation (RMSEA) should score below 0.1 [52,53].

## 3. Results

Table 1 shows the levels of PA, ST, family support and friends’ support in the area of Soria. In addition, the results are displayed according to the gender of the participants.

The suggested model was developed through the variables evaluated in a Spanish sample of high school adolescents and obtained a good fit for each of the different indices of the model. The Chi-Square analysis showed a significant *p*-value (X^2^ = 2.775; df = 1; pl = 0.096), but due to the influence of sample susceptibility and sample size, the data could not be interpreted independently [54], so other standardised fit indices were used. The CFI was a score of 0.995, the NFI was 0.993, the IFI showed a score of 0.995, the TLI showed a score of 0.972, and finally, the RMSEA reflected a score of 0.051.

**Figure 2:**
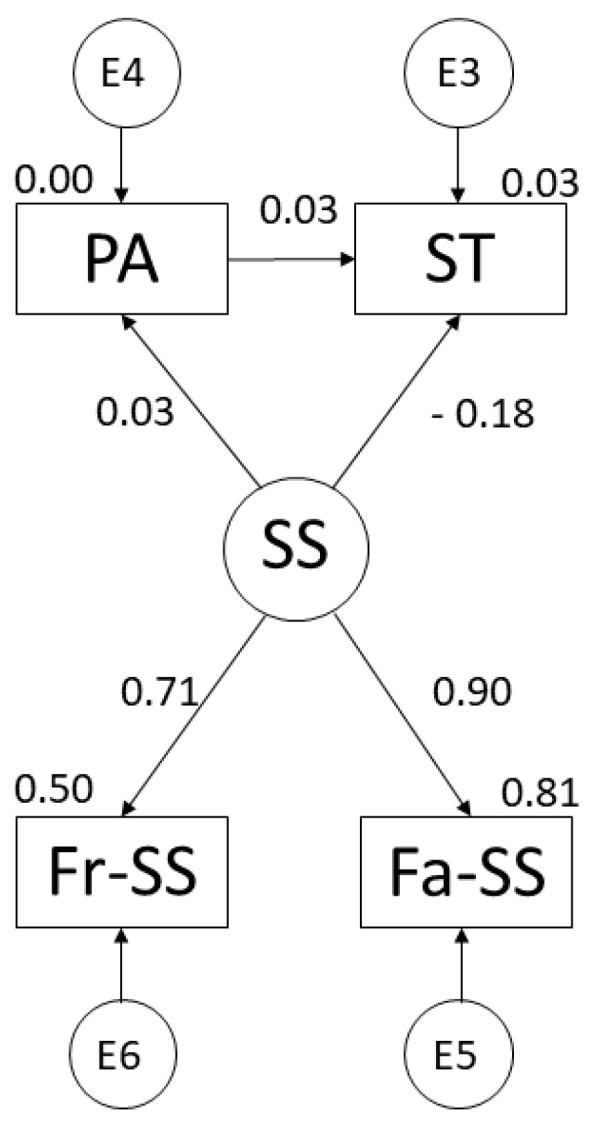
Suggested model for adolescent population. Note: Physical Activity (PA); Social Support (SS); Screen Time (ST); Family Support (Fa-SS); Friends’ Support (Fr-SS).

The regression weights of the theoretical model are shown in Figure 2 and Table 2. Looking at SS, there was a positive relationship with PA practice (r = 0.026), family support (r = 0.901) and friends’ support (r = 0.710; *p* ≤ 0.001) and a negative relationship with ST (r = −0.178; *p* ≤ 0.001). A positive relationship with ST was found for PA (r = 0.028).

Following the model developed for males, a good fit was observed for each of the indices. In this case, the Chi-Square analysis showed a significant *p*-value (X^2^ = 1.231; df = 1; pl = 0.267). The CFI scored 0.999, the NFI reflected a value of 0.993, the IFI showed a score of 0.960, the TLI was 0.992, and the RMSEA reflected a score of 0.025.

**Figure 3:**
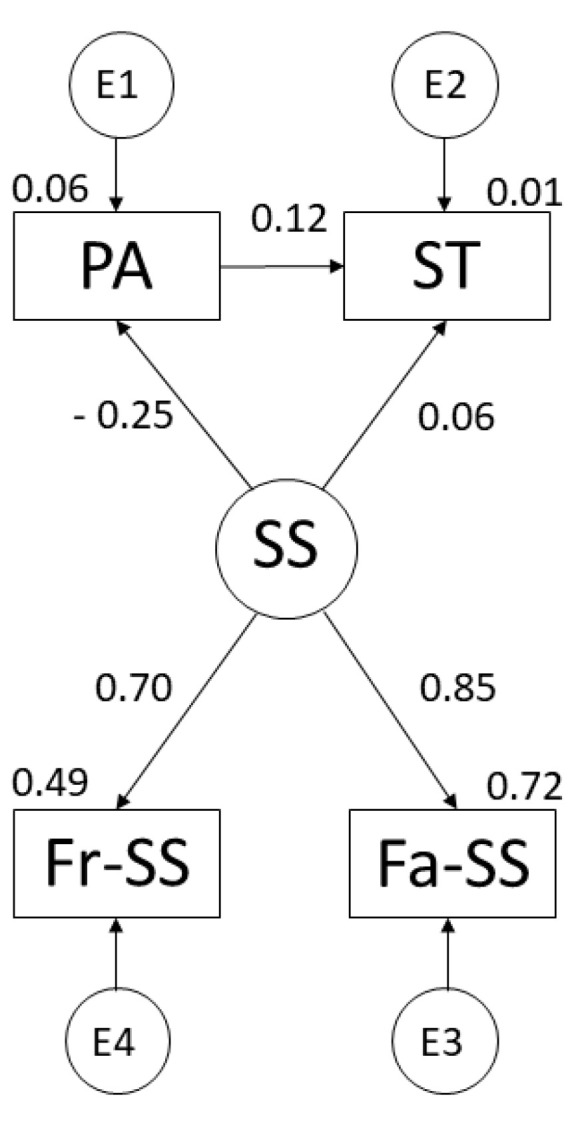
Theoretical model developed for males. Note: Physical Activity (PA); Social Support (SS); Screen Time (ST); Family Support (Fa-SS); Friends’ Support (Fr-SS).

The regression weights of the theoretical model for males are shown in Figure 3 and Table 3. Looking at SS, a positive relationship was observed with family support (r = 0.851), support from friends (r = 0.702; *p* ≤ 0.001) and PA (r = 0.601). However, a negative relationship was shown with ST (r = −0.252; *p* ≤ 0.001). On the other hand, when looking at the relationship between PA and ST, a positive relationship was observed (r = 0.118; *p* ≤ 0.05).

The model for females showed a good fit for each of the different indices. Chi-Square analysis showed a significant *p*-value (X^2^ = 1.480; df = 1; pl = 0.224). The CFI scored 0.998, the NFI reflected a value of 0.993, the IFI showed a score of 0.998, the TLI had a score of 0.986, and finally, the RMSEA scored 0.038.

**Figure 4:**
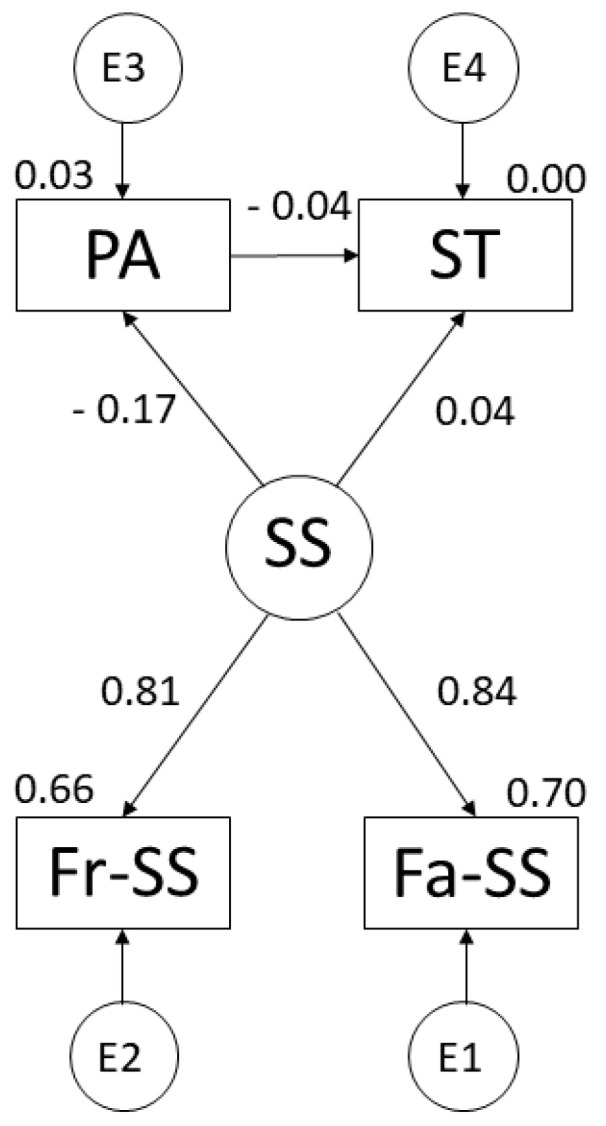
Theoretical model developed for females. Note: Physical Activity (PA); Social Support (SS); Screen Time (ST); Family Support (Fa-SS); Friends’ Support (Fr-SS).

Figure 4 and Table 4 show the regression weights of the theoretical model for females. However, positive relationships were shown with family support (r = 0.839), friendship support (r = 0.813) and PA (r = 0.043). Finally, a negative relationship was observed between ST and PA (r = −0.043).

## 4. Discussion

The current research presented models that explain the relationships between PA, SS and ST in adolescents. The results obtained were in line with the theoretical model initially presented. Following is a discussion of the results found on the basis of the comparison of the explanatory models, relating them to the existing literature.

SS was directly related to PA in a positive but non-significant way in the three models described. This was related to the findings of Mendonça et al. [15], as they did not find a clear predominance, finding two studies in which adolescents had positive relationships between PA and SS, and two others in which there was no relationship. Furthermore, each type of SS seemed to influence PA in different ways [18,55].

Regarding SS for PA, it has been shown that friends are a fundamental agent. In the three models, there was a positive and significant relationship between peers and the latent variable SS. Indeed, a positive and significant relationship between PA practice and perceived peer support was confirmed in the scientific literature [15]. Furthermore, the influence of friends was superior to that of family. This has also been shown in previous studies [16,19,22], just as during childhood, family support is superior to that of friends [17].

Based on Rodrigo et al. [56], other aspects of lifestyle, such as tobacco, alcohol and drug consumption, also showed this changing predominance of SS. At the beginning of adolescence, young people have healthier habits, with high parental support, but later, adolescents acquire less healthy habits, probably due to their need to gain peer acceptance and respect [56].

The models presented for Soria did not show a unique trend in the relationship between PA and ST. For the total sample, the relationship was positive, but not significant. The relationship was positive but also significant in males. On the other hand, in females, the relationship was negative and not significant.

The results obtained for the relationship between PA and ST were different from those of previous studies. O’Brien et al. found a negative relationship between mean daily ST and PA levels. This relationship was non-significant, regardless of PA intensity and adolescent sex [29]. Costigan et al. also found a negative association between ST and PA/fitness in 60% of the studies they selected in their systematic review [57]. These associations were also negative and non-significant for German adolescents aged 13 years in both sexes, but were positive for TV viewing time [33].

The reasons for the differences between the PA and ST ratios described in other studies and the ratios obtained in this one are not known. These may be due to Soria adolescents, particularly the boys, watching TV for long periods of time. This would be similar to the results for German adolescents [33]. Another reason for this difference may be due to the fact that the ST items in the questionnaire used in this study did not ask about the time spent using mobile devices. Although it is not known how this would influence the relationship with PA, it would lead to an increase in ST. This would be due to the fact that adolescents’ time spent on mobile devices is substantial, being higher among females [58] and increasing with the adolescents’ age [40]. In addition, young people spend a significant amount of time on social networks and the internet, especially females [59], activities related to mobile phone use [60].

Furthermore, the differences in the relationship between PA and ST may also be due to the fact that most previous studies did not take other potential factors, such as SS, into consideration alongside. This is in line with the findings of Parker et al. [61]. They observed that SS for PA, both from parents and friends, increased as participant group typologies were characterised by more PA and less sedentary/ST activities. Silva et al. also concluded that SS was a protective factor for the combination of low levels of PA and high levels of ST [62]. In addition, SS for PA from parents and friends predicted ST, PA, moderate PA and vigorous PA of adolescents, with support from friends being a better predictor [16].

In all three models described in this study, the existence of a direct influence of SS towards ST was demonstrated. In all three cases, the relationship was negative, weak and significant. These results follow trends in other studies. For example, Costigan et al. [57] found that 75% of the studies on the topic showed a negative association between screen-based sedentary behaviour and SS/socialising in adolescent females. Park and Park also found a similar link in an explanatory model of relationships between parental SS for PA, PA, ST and body weight in U.S. adolescents [63].

The study conducted in Soria has several limitations. One of them is related to the instruments used. For measuring PA levels, other more reliable instruments, such as accelerometers or pedometers, could have been used. Another limitation is related to the ST activities. The PA questionnaire asked about ST activities, such as watching TV and the use of “computer, video games and internet”, but did not take into account the time spent using mobile phones or tablets. This would have led to an increase in adolescents’ ST. A third limitation is related to the type of study conducted. Being cross-sectional, it served to analyse the relationships between the variables studied in a specific period of time. Moreover, the results cannot be generalised to other places, as the adolescents were exclusively from the city of Soria. Finally, there is a limitation derived from the type of non-probability sampling. It was decided to use this sampling because more participants were available than were considered statistically sufficient, that is, for an error of precision of 5%. For this reason, and because at least one group of students was selected from all of the centres that wished to participate in the study, this type of sampling was preferred, and the error of precision was substantially reduced (3.3%).

To conclude, we would like to highlight some future lines of research. It would be advisable to carry out longitudinal studies on the research topic, in order to find out how the explanatory relationships of the models vary over time: for example, to identify variations in the influence of social support as the age of the participants increases. Moreover, these studies would be more complete if participants over a wider age range, such as children, adolescents and adults, were included. It would also be possible to detail models in which different types of SS are set as variables, in order to study their relationships with PA and ST in a more concrete way. In addition, intervention programs could be designed and implemented to increase PA levels of adolescents by including friends as participants, independent of other variables such as BMI, sex, grade, or class group membership.

## 5. Conclusions

It has been shown that the theoretical model initially presented was useful to explain the relationships between PA, SS and ST of teenagers from Soria (Spain). Moreover, this model was useful in explaining these relationships according to participants’ sex, since acceptable values were obtained for the parameters of the general equation.

SS for PA practice was shown to be a determinant of adolescents’ ST. In particular, the influence of perceived support from friends was noteworthy.

There was no clear trend in the relationship between PA and ST. The overall model showed a positive relationship. However, according to sex, in the males’ model, it was positive and significant and in the females’ model, it was negative and non-significant.

Furthermore, in the models shown, SS had a positive but non-significant relationship with PA.

## Figures and Tables

**Table 1:** Descriptive characteristics of the variables.

Variable	Total (M ± SD)	Gender (M ± SD)	
		Male	Female
Physical Activity	893.88 ± 57.54	891.64 ± 55.92	896.35 ± 59.27
Screen Time	117.70 ± 76.69	136.93 ± 81.55	96.49 ± 64.74
Family Support	2.09 ± 0.81	2.15 ± 0.82	2.03 ± 0.80
Friends’ Support	1.72 ± 0.60	1.78 ± 0.60	1.65 ± 0.60

**Table 2:** Structural model of the theoretical model.

Variable Associations	R.W.				S.R.W.
	Estimations	S.E.	C.R.	*p*	Estimations
ST←SS	−18.699	5.517	−3.390	***	−0.178
Fa-SS←SS	1.000				0.901
Fr-SS←SS	0.584	0.136	4.306	***	0.710
PA←SS	2.072	3.279	0.632	0.527	0.026
ST←PA	0.038	0.050	0.755	0.450	0.028

Note: Regression Weights (R.W.); Standardised Regression Weights (S.R.W.); Standard Error (S.E.); Critical Ratio (C.R.); Screen Time (ST); Social Support (SS); Family Support (Fa-SS); Friends’ Support (Fr-SS); Physical Activity (PA); *** *p* ≤ 0.001.

**Table 3:** Structure of the theoretical model for males.

Variable Associations	R.W.				S.R.W.
	Estimations	S.E.	C.R.	*p*	Estimations
ST←SS	−29.565	8.506	−3.476	***	−0.252
Fa-SS←SS	1.000				0.851
Fr-SS←SS	0.604	0.145	4.154	***	0.702
PA←SS	4.932	5.011	0.984	0.325	0.601
ST←PA	0.081	0.037	2.162	**	0.118

Note: Regression Weights (R.W.); Standardised Regression Weights (S.R.W.); Standard Error (S.E.); Critical Ratio (C.R.); Screen Time (ST); Social Support (SS); Family Support (Fa-SS); Friends’ Support (Fr-SS); Physical Activity (PA); *** *p* ≤ 0.001; ** *p* ≤ 0.05.

**Table 4:** Structure of the theoretical model for females.

Variable Associations	R.W.				S.R.W.
	Estimations	S.E.	C.R.	*p*	Estimations
ST←SS	−16.622	6.432	−2.584	**	−0.173
Fa-SS←SS	1.000				0.839
Fr-SS←SS	0.722	0.216	3.339	***	0.813
PA←SS	3.807	5.512	0.691	0.490	0.043
ST←PA	−0.040	0.051	−0.0770	0.441	−0.043

Note: Regression Weights (R.W.); Standardised Regression Weights (S.R.W.); Standard Error (S.E.); Critical Ratio (C.R.); Screen Time (ST); Social Support (SS); Family Support (Fa-SS); Friends’ Support (Fr-SS); Physical Activity (PA); *** *p* ≤ 0.001; ** *p* ≤ 0.05.

## Data Availability

Not applicable.
